# Use of prophylactic uterotonics during the third stage of labor: a survey of provider practices in community health facilities in Sierra Leone

**DOI:** 10.1186/s12884-016-0809-z

**Published:** 2016-01-28

**Authors:** Abirami Natarajan, Roy Ahn, Brett D. Nelson, Melody Eckardt, Jennifer Kamara, SAS Kargbo, Pity Kanu, Thomas F. Burke

**Affiliations:** Division of Global Health and Human Rights, Department of Emergency Medicine, Massachusetts General Hospital, Zero Emerson Place Suite 104, Boston, MA 02114 USA; Harvard Medical School, 25 Shattuck Street, Boston, MA 02115 USA; Ministry of Health and Sanitation, 4th Floor Youyi Building, Freetown, Sierra Leone

**Keywords:** Prophylactic uterotonics, Postpartum hemorrhage, Sierra Leone

## Abstract

**Background:**

Postpartum hemorrhage remains the leading cause of maternal mortality worldwide. Administration of uterotonics during the third stage of labor is a simple and well established intervention that can significantly decrease the development of postpartum hemorrhage. Little is known about the use of prophylactic uterotonics in peripheral health centers, where the majority of normal deliveries occur. The purpose of this study is to assess health provider current practices and determinants to the use of prophylactic uterotonics in Sierra Leone, a country with one of the highest maternal mortality ratios worldwide.

**Methods:**

This is a mixed methods study using descriptive cross-sectional survey and qualitative interviews in community health facilities in Freetown, Sierra Leone following a comprehensive training on postpartum hemorrhage. Facilities and providers were surveyed between May and June 2014. Qualitative methods were used to identify barriers and facilitators to the use of prophylactic uterotonics.

**Results:**

A total of 134 providers were surveyed at 39 periphreal health facilities. Thirteen facilities (39 %) reported an inconsistent supply of oxytocin. The majority of facilities (64 %) stored oxytocin at room temperature. Provider level, in-service training, and leadership role were significantly associated with prophylactic uterotonic use. Overall, 62 % of providers reported routine use. Midwives were most likely to routinely administer uterotonics (93 %), followed by community health officers/assistants (78 %), maternal and child health aides (56 %), and state-enrolled community health nurses (52 %). Of the providers who received in-service training, 67 % reported routine use; of those with no in-service training, 42 % reported routine use. Qualitative analysis revealed that facility protocols, widespread availability, and provider perception of utility facilitated routine use. Common barriers reported included inconsistent supply of uterotonics, lack of knowledge regarding timely administration, and provider attitude regarding utility of uterotonics following normal deliveries.

**Conclusion:**

There is considerable room for improvement in availability and administration of prophylactic uterotonics. Understanding barriers to routine use may aid in developing multifaceted pre-service and in-service training interventions designed to improve routine intrapartum care.

## Background

Postpartum hemorrhage (PPH) is the most common cause of maternal mortality and morbidity worldwide, with the majority of PPH-related deaths occurring in low-income countries [1–3]. Active management of the third stage of labor (AMTSL) is a well-established protocol that has been shown to significantly reduce the incidence of PPH [4, 5]. AMTSL consists of administration of a prophylactic uterotonic after delivery of the newborn, fundal massage, delayed cord clamping and controlled cord traction. The initial studies evaluating the efficacy of AMTSL studied these components as a package. Although there is strong evidence to recommend the use of prophylactic uterotonic for the prevention of PPH, the relative importance of controlled cord traction and fundal massage is uncertain [6, 7]. Based on this, the International Federation of Gynecologists and Obstetricians (FIGO) and the World Health Organization (WHO) recommend routine use of uterotonics to prevent PPH [8, 9].

Despite strong evidence supporting the effectiveness of these guidelines, there remains significant inter-country variation in rates of AMTSL uptake. A WHO cross-sectional survey of 15 university-based obstetric centers in both developing and developed countries found low observed use of AMTSL, occurring on average in only 25 % of deliveries [10]. Other studies investigating both AMTSL practices and interventions targeted at improving AMTSL adherence in the hospital setting have also found low rates of knowledge and application [11–14].

Most research has understandably focused on high-volume facilities or complicated deliveries; however, little is known about AMTSL practices in primary health facilities in particularly low income countries, where normal uncomplicated deliveries are encouraged [15]. These facilities are fundamentally different from hospitals in that they employ lower-skilled providers, have limited access to health information, and have fewer resources to manage the consequence of PPH. However, given the role of preventive practices, understanding provider adherence to interventions that prevent PPH at these facilities can provide insight into opportunities for improving routine intrapartum care – a cornerstone strategy for addressing maternal mortality [16].

The purpose of this study is to gain further insight into provider use of prophylactic uterotonics following a comprehensive PPH-training package in Freetown, Sierra Leone. We sought to understand the personal practice of prophylactic uterotonic administration in providers conducting routine deliveries. Furthermore, we sought to understand if factors such as in- service AMTSL training, provider cadre, and leadership positions were associated with the routine use of uterotonics. In addition, qualitative methods were used to identify facilitators and barriers to routine use of prophylactic uterotonics.

## Methods

In December 2013, Massachusetts General Hospital in conjunction with the Sierra Leone Ministry of Health conducted a total of eight three-hour workshops on a PPH in-service training called Every Second Matters for Mothers and Babies – Uterine Balloon Tamponade (ESM-UBT). The training components of ESM-UBT included AMTSL (administration of 10 IU oxytocin IM or IV, fundal massage, delayed cord clamping and controlled cord traction), basic PPH management, and the use of condom-catheter uterine balloon tamponade (UBT) as a second-line treatment for uncontrolled PPH [17]. Two representatives from each of 46 peripheral health facilities – usually a facility head and an experienced midwife – were asked to attend this session and subsequently disseminate the knowledge to all members of the facility who are involved in conducting deliveries. Following the central training, these representatives – referred to as “UBT champions” - were contacted via phone to ensure that all members of the facility were trained. Each facility was provided with two PPH instruction manuals, a pictorial wall chart, and condom-catheter UBT kits. By March 2014, all of the original facility representatives reported that all members of their facility had been trained.

This was a descriptive cross-sectional survey of reported uterotonic use during the third stage of labor for providers working in peripheral health care facilities. This survey was embedded within a larger qualitative study assessing facilitators and barriers to uptake of the ESM-UBT training package (results reported separately). During May – June 2014, approximately 6 months after the central training session, a survey was conducted at 39 of the 42 (92.9 %) peripheral health facilities that attended the initial central training. Three facilities were not visited because of difficult terrain.

At each facility, information regarding the availability and storage of uterotonics was obtained from facility or maternity heads. Since oxytocin is the primary uterotonic used and freely distributed by the Sierra Leone government, providers were specifically asked if they had a consistent supply of oxytocin available at their facility. The heads of the facility were then asked to identify all members of the facility who conduct deliveries on that particular shift. All providers who conduct deliveries in primary health facilities in Sierra Leone are authorized to use oxytocin during the third stage of labor per the Sierra Leone national protocol for the prevention and treatment of PPH. Each provider was asked general questions about their professional training, in-service training in PPH (including AMTSL), years of experience, and delivery volume. Providers were then asked about their personal practice of managing the third stage of labor during vaginal deliveries. Qualitative interviews were conducted to capture provider practices, attitudes, and perceived barriers to routine use of prophylactic uterotonics. Verbal informed consent was obtained from all participants. All data were recorded and entered into a Microsoft Excel spreadsheet. Clinical results are reported as mean ± SEM. Analyses between groups for statistically significant differences in categorical data were performed with the Fisher’s Exact and Fisher-Freeman-Halton Tests. Student’s t-tests were used to assess differences among groups with continuous variables (R version 3.1.1).

Ethical approval for this study was obtained from the Partners Healthcare Human Research Committee (12/18/2013 protocol#: 2012P002112/MGH) and Sierra Leone Ministry of Health and Sanitation (05/12/2014).

## Results

A total of 134 providers were surveyed at 39 study sites (1–8 interviews per site). Table 1 shows the characteristics of health facilities, including average delivery volume, oxytocin availability, and oxytocin storage. The majority of facilities (62 %) were CHCs, followed by CHPs (26 %), and MCHPs (13 %). Oxytocin was most consistently available at MCHPs (80 %), followed by CHP (60 %), and CHCs (58 %). Fifteen facilities (44 %) reported shortages of oxytocin. The majority of facilities (64 %) reported storing oxytocin at room temperature.

**Table 1:** Characteristics of health facilities

Facility type (*n* = 39)	Average number of deliveries in facility/month	Oxytocin availability (% always available)	Oxytocin storage (% stored in fridge)
MCHP (*n* = 5)	21	4 (80 %)	0 (0.0 %)
CHP (*n* = 10)	31	6 (60 %)	2 (20 %)
CHC (*n* = 24)	50	14 (58 %)	11 (46 %)

*CHP* community health post, *MCHP* maternal and child health post, *CHC* community health center

Table 2 shows the characteristics of providers interviewed. Providers reported conducting between 1 and 100 deliveries per month with a mean (SD) of 20 (19.2) deliveries per month. Their experience ranged from 6 months to 32 years, with a mean (SD) of 7.2 (6.7) years. The majority of respondents was female (90 %). MCHAs (51 %) and SECHNs (25 %) accounted for the majority of respondents. Thirteen providers (21 %) held administrative positions – either head of facility or maternity ward. The majority of providers (77 %) received targeted training in AMTSL since their professional training.

**Table 2:** Characteristics of obstetric providers interviewed

Characteristics	Number	Percent (%)
Experience (years)		
0-9	97	72
10-19	27	20
20-29	6	5
>30	4	3
Gender		
Male	13	10
Female	121	90
Cadre		
Midwife	14	11
SECHN	34	25
MCHA	68	51
CHO/CHA	18	13
Leadership*		
Yes	28	
No	106	21
AMTSL training		79
In-service AMTSL training	103	77
No in-service training	31	23
Provider average deliveries/month		
1-19	81	60
20-39	32	24
40-59	13	10
60-79	5	4
80-100	3	2

*Leadership position includes providers in charge of the facility or in charge of the labor and delivery room

When providers were asked about their management of the third stage of labor, 83 (62 %) of the 134 providers reported that they routinely give prophylactic uterotonics. With the exception of one provider who used misoprostol, all providers who reported uterotonic use reported using oxytocin. The relationship between provider characteristics and reported use of uterotonics is outlined in Table 3. Provider level, in-service training, leadership roles, and years of experience were significantly associated with reported use of uterotonics (p < 0.05). Midwives were most likely to administer oxytocin (93 %), followed by CHO/CHA (78 %), MCHAs (56 %), and SECHNs (53 %). Providers who received a variation of in-service training were more likely to report administration of uterotonics that those who did not (p < 0.01). Of the 103 providers who had in-service training, 69 (67 %) gave uterotonics. Less than half (42 %) of the 31 providers who did not receive in-service training administered uterotonics during the third stage of labor. Providers who administered uterotonics on average had 8.5 years of experience (SEM 0.87) compared with those who did not report using uterotonics, who had 5.1 (SEM 0.43) years of experience.

**Table 3:** Relationship between provider characteristics and reported routine administration of prophylactic uterotonics

Provider level of training	Number (percentage) who report routinely administered prophylactic uterotonic	*P* < 0.05
MCHA	38/68 (56 %)	
SECHN	18/34 (53 %)	
Midwife	13/14 (93 %)	
CHO/CHA	14/18 (78 %)	
Total	83/134 (62 %)	
Type of training		*P* < 0.01
In service training	69/103 (67 %)	
No in-service training	13/31 (42 %)	

Interviews with facility leaders, maternity heads, and providers revealed facilitators and barriers to routine uterotonic use during the third stage of labor (representative quotations shown in Table 4). Established facility protocols, perception of prophylactic uterotonics as a lifesaving drug, and a clear understanding of its indication facilitated routine administration. Several providers reported that oxytocin supplied by the government was both insufficient and inconsistently available at their facility; however, providers who perceived it as an essential and lifesaving drug strongly encouraged patients and their families to purchase oxytocin prior to giving birth. Several providers – usually facility heads and experienced midwives – reported using personal funds to purchase oxytocin during government supply shortages. The majority of these providers expressed being motivated by an understanding that not providing prophylactic uterotonics would be providing suboptimal care to a patient. A very small number of providers also expressed fear of being audited by the government in the case of a poor maternal outcome as a driver to purchasing backup supplies.

**Table 4:** Representative quotes regarding facilitators and barriers to the use of prophylactic uterotonics during the third stage of labor

Facilitators
Providers who understood the value of prophylactic uterotonics as a life saving drug purchased oxytocin when the government supply was inconsistent.
• “Because oxy is an emergency drug it is very important for delivery. Sometime it is short, but I buy and keep it in the cupboard. Yea but sometime government have shortage and don’t give us, but we buy.” – Clinical Health Officer • “I want to save life, I’m not going to depend on government to give me oxy, so that’s why we usually buy it, whenever we have a case. “– Midwife
Established protocols in facility for preventing PPH
• “One of the protocols is that when the woman has delivered we give oxytocin.... All of this management is an attempt to prevent PPH.” – Clinical Health Officer
Providers who understand the importance of oxytocin as a life saving drug strongly encouraged patients to purchase oxytocin when it was unavailable
• “When we are short of oxytocin, we buy. If there is nothing, we tell them [patients], when they are term, birth preparedness, this will one of the things that will be in the kit. You bring this, you bring this, and you bring this. The nurses write for them to bring. But normally it is around. For those who can’t afford, we give. “– Clinical Health Officer • “We ask them [patients], we force them to buy it because you know after delivery we need to help them with oxytocin.” – midwife
Providers purchase oxytocin out of fear of being audited
• “They order it so that we can save our own selves, because if you have a maternal death and you are on duty, they will query you. They will judge you and you have to prove yourself, you have to take all of your documents and explain what happened and what did not happen. So you will not allow that. So even if the midwife is not around, we have to take it out of [our own money].” – Maternal and Child Health Aid
Barriers
Providers reported giving uterotonics when it was available, but during shortages managed the third stage expectantly.
• “We give oxytocin if we have, but if we don’t have then we express normal procedures [expectant management].” – MCHA
Misconceptions about the universal indication of uterotonics – such as prolonged labor or if there is difficulty delivering placenta - prevented providers from giving routinely
• “At times – we do not give oxytocin if the placenta comes out easily. There is no need to give oxytocin.” • “Only when it is difficult labor we are told to give. But we haven’t had any yet.
Some providers report learning that uterotonics should be given to all patients but believe that it is not required unless the mother is bleeding
• “Well because we are supposed to give, but when we don’t have the case and we are not seeing enough profuse bleeding, I do not see justification to give. So normally we do fundal massage for contractions to take place, and if there are clots then we expel. We check for tear, perineal, cervical and we see how best – based on that if there is no profuse bleeding we don’t give.” – Clinical Health officer
Belief that prophylactic oxytocin is not needed if a patient delivers normally
• “After the delivery I didn’t give oxytocin because she delivered normally. Only if she is bleeding. Because some of them they deliver normally, no problem.” – MCHA • “After delivery I do not give any oxytocin because she delivered normally. Later on after 30–40 min {inaudible} so I had to set my normal saline with 20 IU oxytocin and the woman was still bleeding so I called for sister to help me.” - MCHA

Barriers to the consistent use of prophylactic uterotonics included inconsistent supply and correct knowledge regarding proper indications for administration. Providers expressed significant concern when oxytocin was unavailable at their facility. Some providers reported the use of uterotonics during the third stage of labor when it was available, but resorted to expectant management when the government supply of uterotonics was unavailable and patients could not purchase it due to either cost or access. Gaps in knowledge regarding the proper indication and utility of prophylactic uterotonics prevented providers from its routine administration. Several providers expressed their belief that women who underwent normal labor did not require prophylactic uterotonics following delivery of the newborn. Other providers specified that they would provide prophylactic uterotonics if a patient’s labor was prolonged, there were challenges delivering the placenta, or if there was any bleeding during or immediately after delivery. Several providers reported that they were taught both in their traditional and in-service training to give prophylactic uterotonics routinely to all women during the third stage of labor, but they did not see a justification for this if a woman was not bleeding, and therefore did not routinely administer it.

## Discussion

Our results showed that although there is a widespread awareness, acceptance, and use of prophylactic uterotonics in Sierra Leone, there remains considerable room for improvement. Of the134 providers surveyed, 62 % reported routine use of uterotonics during the third stage of labor. Although all health cadres are taught AMTSL during pre-service training, those who received in-service training including AMTSL were significantly more likely to administer prophylactic uterotonics than those who did not.

Providers in leadership positions such as CHOs and midwives were also more likely to report uterotonic use than providers who are frontline in conducting normal uncomplicated deliveries (SECHNs and MCHAs). Ninety-three percent of midwives and 77 % of CHOs/CHAs reported routine use of uterotonics. These findings may reflect differences in initial training or provider level of experience. Unfortunately, providers with more experience such as midwives and CHOs/CHAs are more likely to be involved after PPH has already developed, rather than during the routine management of labor. This finding, however, does represent a strong basis for transfer of knowledge within a facility. Quality improvement interventions focused on the active guideline development by facility leaders – which has shown promise in other settings – may play a role in increasing routine uterotonic use [18].

Consistent provision of drugs is required for the timely administration of any intervention. Although oxytocin is an essential drug freely supplied by the Sierra Leone government, providers cite that inadequate supply of uterotonics remains a significant challenge to routine administration. Our results showed that 39 % of facilities had an inconsistent supply of oxytocin. Furthermore, only one-third of facilities stored oxytocin within the manufacturer-recommended temperature of 2–8°. Although providers who perceived the value of oxytocin purchased it with their own funds or strongly encouraged patients to do so, many providers reported that oxytocin shortages contributed to their use of expectant management of the third stage of labor. Limited availability of oxytocin in primary health facilities likely also has implications for the emergent treatment of postpartum hemorrhage. Further research is needed to assess the efficacy of oxytocin in room temperature conditions as well as the role of temperature independent uterotonics such as misoprostol in resource limited settings.

### Study strengths and limitations

The major strength of this study is the location. Given that Sierra Leone carries one of the highest maternal mortality ratios worldwide, understanding provider practices of preventive interventions in peripheral health facilities where routine deliveries are encouraged serves as a basis for targeted quality improvement. In addition, few studies have documented specific provider perceptions that remain barriers to routine prophylactic uterotonic use. Limitations of this study include its descriptive nature and small sample size. Interviews are subject to recall, and given desirability bias, providers may over-report their use of oxytocin. Furthermore, this study did not assess the timing of oxytocin administration. Given that uterotonics are recommended within one minute after delivery of the newborn, it is likely that observed deliveries in these facilities would be less than optimal. Observed studies conducted in high-volume hospitals have indeed found a discrepancy between provider knowledge and timely administration of prophylactic uterotonics [19]. However, observing deliveries in peripheral facilities with lower deliveries presents logistical and financial challenges.

## Conclusion

Despite evidence-based guidelines recommending the use of prophylactic uterotonics during the third stage of labor, hospitals in both developed and developing countries significantly underuse it. This study documents provider practices administering prophylactic uterotonics in lower-level facilities in Sierra Leone, and provides insight into several underlying reasons for its underutilization. Although 67 % of providers who underwent in-service training reported the use of uterotonics, there still remains a gap in optimal translation from in-service training to knowledge retention and clinical practice. Other studies have documented this, as well as the need for multiple components in addition to dissemination of knowledge for clinical behavior change to occur [11, 12]. Further research is needed to determine the optimal duration, components, and delivery of targeted AMTSL training.

Among the providers who did not routinely administer uterotonics, supply of drugs, provider knowledge of proper indication, and provider attitudes toward the utility of prophylactic uterotonics were the most common barriers to routine administration. For instance, several providers described their belief that mothers who underwent normal deliveries or did not have bleeding did not require prophylactic uterotonics. This belief may lead to sporadic use of prophylactic uterotonics, particularly in the context of drug shortages. Professional and in-service training presents an opportunity to emphasize and reinforce the value of routine administration of uterotonics even in normal deliveries.

There remains considerable room for quality improvement in administering prophylactic uterotonics during the third stage of labor. Although inconsistent supply of uterotonics remains a barrier to routine use, accurate provider knowledge regarding indications for and value of prophylactic uterotonics also contribute to its underutilization. Emphasis on incorporating and emphasizing the use of uterotonics during pre-service and in- service training and the development of facility protocols may improve practices. Consistent availability and implementation of oxytocin during the third stage of labor represents a very simple, feasible, inexpensive intervention to decrease bleeding postpartum, the most common cause of maternal mortality worldwide.

## Abbreviations

AMTSL: Active management of the third stage of labor
PPH: Post partum hemorrhage
UBT: Uterine balloon tamponade
SECHN: State enrolled child health nurses
MCHA: Maternal and child health aides
CHO: Clinical health officers
FIGO: International Federation of Gynecologists and Obstetricians
ICM: International Confederation of Midwives
WHO: World Health Organization
